# Supplementary material for: Controllable skyrmion chirality in ferroelectrics
Source: Sci Rep. 2020 May 26;10:8657. doi: 10.1038/s41598-020-65291-8 (PMC7251125; doi:10.1038/s41598-020-65291-8)
Supplement: Supplementary file 1 — Supplementary Information. [file 41598_2020_65291_MOESM1_ESM.pdf]

# SUPPLEMENTARY INFORMATION for:
# Controllable skyrmion chirality in ferroelectrics

Yu. Tikhonov,[1,2] S. Kondovych,[2,3] J. Mangeri,[4,5] M. Pavlenko,[1] L. Baudry,[6] A. Sené,[2] A. Galda,[7] S. Nakhmanson,[8,5] O. Heinonen,[9] A. Razumnaya,[1] I. Luk'yanchuk,[10,11] and V. M. Vinokur[9,12]

[1]*Faculty of Physics, Southern Federal University, 5 Zorge str., 344090 Rostov-on-Don, Russia.*

[2]*University of Picardie, Laboratory of Condensed Matter Physics, Amiens, 80039, France.*

[3]*Life Chemicals Inc., Murmanska st. 5, Kyiv, 02660, Ukraine.*

[4]*Institute of Physics, Academy of Sciences of the Czech Republic, Na Slovance 2, 18221 Praha 8, Czech Republic.*

[5]*Department of Physics, University of Connecticut, Storrs, CT, USA.*

[6]*Institute of Electronics, Microelectronics and Nanotechnology (IEMN)-DHS Départment, UMR CNRS 8520, Université des Sciences et Technologies de Lille, 59652 Villeneuve d'Ascq Cedex, France.*

[7]*James Franck Institute, University of Chicago, Chicago, Illinois 60637, USA.*

[8]*Department of Materials Science & Engineering and Institute of Material Science, University of Connecticut, Storrs, Connecticut 06269, USA.*

[9]*Materials Science Division, Argonne National Laboratory, 9700 S. Cass Avenue, Argonne, Illinois 60637, USA.*

[10]*University of Picardie, Laboratory of Condensed Matter Physics, Amiens, 80039, France;*

[11]*Landau Institute for Theoretical Physics, Akademika Semenova av., 1A9 Chernogolovka, 142432, Russia.*

[12]*Consortium for Advanced Science and Engineering (CASE) University of Chicago, 5801 S Ellis Ave, Chicago, IL 60637, USA.*

**Phase-field simulations.**

The phase-field simulations have been carried out in the framework of open source FERRET package [1], developed for the open source multi-physics simulation environment MOOSE [2]. After variation of free-energy functional $F$ (equation (2) in paper) with respect to polarization components $P_i$ ($i = 1, 2, 3$)

$$-\gamma \frac{\partial P_i}{\partial t} = \frac{\delta F}{\delta P_i}, \tag{1}$$

we obtain actual equations to expose to finite element computation scheme. The relaxation parameter $\gamma$ setting the fictitious time scale for the computational procedure, was taken equal to unity since this fictitious time scale does not influence the free energy minima distribution in our problem.

Supplementary Fig. S1 depicts the conceptual simulation space exposing the details of the generated finite-element grid. The computational domain $\Omega$ is a cylindrical medium $\Omega_m$ embracing the ferroelectric nanodot $\Omega_d$. The total surface of $\Omega_m$, i.e. that of $\Omega$ is defined as $\partial\Omega_m \cup \partial\Omega_t \cup \partial\Omega_b$, where $\partial\Omega_m$ is side surface of $\Omega_m$ and $\partial\Omega_t$, $\partial\Omega_b$ are the top and bottom surfaces of $\Omega_m$, respectively. The total surface of the nanodot is denoted as $\partial\Omega_d$. An open source 3D finite element mesh generator gmsh [3] was used to generate the finite element meshes representing the computational domain. We used the non-structured meshes based on the tetrahedral elements. The variable density of elements lowering from the surface of the nanodot towards the surface of the medium results in the faster and less in volume consuming memory (both the storage and the random-access) consuming computations and does not reduce the precision of the results.

The variational formulation of problem was transformed into the weak form [4] for which the unknowns polarization $P$ and the electric potential $\varphi$ are functions of the $H^1(\Omega)$ space. The solution for the discreticized variational problem was sought in the form of $P_1$ Lagrange finite elements. The voltage applied to the electrodes was introduced into the problem as boundary conditions of the Dirichlet type at the $\partial\Omega_t$ and $\partial\Omega_b$ imposed on unknown $\varphi$. The Newton-Raphson method coupled with the generalized minimal residual method (GMRES) and block Jacobi preconditioner [5, 6] was used to solve the resulting system of the non-linear algebraic equations. When solving the partial differential equations which depend on time, one needs to choose the appropriate time discretization technique and the initial conditions for the polarization distribution. We used the 2-step backward differentiation formula to implement the time-stepping scheme with the random distribution of individual polarization components varying at each node of the finite-element mesh from $-10^{-5}$ to $10^{-5}$ C/m$^2$ at the initial time step in the case of the simu-

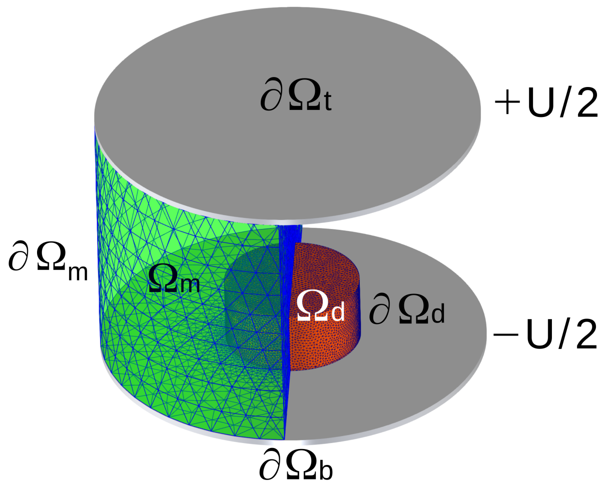


**Supplementary Figure S1. Simulation setup.**

lation at the point 0 and the distribution acquired as the previous solution in all other points of the hysteresis curve. The criteria for the terminating the solution process at each point is $|(|\mathcal{F}_n| - |\mathcal{F}_{n-1}|)/|\mathcal{F}_n|| < 10^{-6}$, where $\mathcal{F}$ is the full energy of system defined by the integral of Eq. (2) of the main text divided by $\Omega_d$.

---

[1] Mangeri, J., Espinal, Y., Jokisaari, A., Alpay, S. P., Nakhmanson, S., and Heinonen, O. Topological phase transformations and intrinsic size effects in ferroelectric nanoparticles. *Nanoscale* **9**, 1616-1624 (2017).

[2] Gaston, D., Newman, C., Hansen, G., and Lebrun-Grandie, D. Moose: A parallel computational framework for coupled systems of nonlinear equations. *Nucl. Eng. and Design* **239**, 1768-1778 (2009).

[3] Geuzaine, C., and Remacle, J.-F. Gmsh: A 3-d finite element mesh generatorwith built-in pre- and post-processing facilities. *Int. Journ. for Numerical Methods in Engineering* **79**, 1309-1331 (2009).

[4] Brenner, S. C., Scott, L. R. *The Mathematical Theory of Finite Element Methods*, Springer, 3rd edition (2008).

[5] Abhyankar, S., Brown, J., Constantinescu, E. M., Ghosh, D., Smith, B. F., and Zhang, H. PETSc/TS: A modern scalable ODE/DAE Solver Library. Preprint arXiv:1806.01437 (2018).

[6] Balay, S., Gropp, W. D., McInnes, L. C., and Smith, B. F., Efficient managementof parallelism in object oriented numerical software libraries. In Arge, E., Bruaset, A. M., Langtangen H. P., editors, *Modern Software Tools in Scientific Computing*, 163-202, Birkhäuser Press (1997).
