# Supplementary material for: Controllable skyrmion chirality in ferroelectrics
Source: Sci Rep. 2020 May 26;10:8657. doi: 10.1038/s41598-020-65291-8 (PMC7251125; doi:10.1038/s41598-020-65291-8)
Supplement: Supplementary file 2 — Supplementary Video Caption. [file 41598_2020_65291_MOESM2_ESM.pdf]

Video description:
Left panel: Animation of the evolution of the polarization states upon poling the nanodot from the zero-field state along the virgin curve (the gray branch in Fig. 2d) and then by the repolarization of the nanodot from the up- to down- oriented polarized state (the blue branch in Fig. 2d). The view is from the bottom of the nanodot. The blue and red points and yellow arrows correspond to the up-, down- and side-oriented polarization. The distribution of the chirality is shown as a color map.
Right Panel. The moving dot in the chirality-field dependence (Fig. 2d) indicates the location of the currently visualized polarization state.
